# PD-L1高表达晚期非小细胞肺癌患者单纯免疫治疗与免疫联合化疗疗效比较

**DOI:** 10.3779/j.issn.1009-3419.2021.103.02

**Published:** 2021-03-20

**Authors:** 浩洋 李, 娜 秦, 孟军 俞, 丽 马, 羽华 吴, 卉 张, 新勇 张, 曦 李, 敬慧 王

**Affiliations:** 1 101149 北京，北京市结核病胸部肿瘤研究所，首都医科大学附属北京胸科医院肿瘤内科 Department of Medical Oncology, Beijing Tuberculosis and Thoracic Tumor Research Institute, Beijing Chest Hospital, Capital Medical University, Beijing 101149, China; 2 101149 北京，北京市结核病胸部肿瘤研究所，首都医科大学附属北京胸科医院肿瘤研究中心 Cancer Research Center, Beijing Tuberculosis and Thoracic Tumor Research Institute, Beijing Chest Hospital, Capital Medical University, Beijing 101149, China

**Keywords:** 免疫检查点抑制剂, 肺肿瘤, 程序性死亡受体-配体1, 单药治疗, 联合治疗, Immune checkpoint blockade, Lung neoplasms, Programmed cell death-ligand 1, Monotherapy, Combination chemotherapy

## Abstract

**背景与目的:**

以免疫检查点抑制剂（immune checkpoint inhibitors, ICIs）为代表的免疫治疗越来越广泛地应用于肺癌治疗。然而，对于程序性死亡受体配体1（programmed cell death-ligand 1, PD-L1）高表达，即肿瘤比例评分（tumor proportion score, TPS）≥50%的晚期非小细胞肺癌（non-small cell lung cancer, NSCLC）患者，采用单纯免疫治疗还是免疫联合化疗在临床上仍存争议。本研究旨在评估PD-L1高表达的晚期NSCLC患者接受单纯免疫治疗与免疫联合化疗的疗效。

**方法:**

本研究回顾性分析了49例PD-L1高表达晚期NSCLC患者的临床资料。PD-L1表达采用22C3抗体行免疫组化染色，按TPS判读PD-L1表达水平。比较不同临床特征分组患者的客观缓解率（objective response rate，ORR）和无进展生存时间（progression free survival, PFS）。

**结果:**

免疫单药与免疫联合化疗组的ORR分别为47.1%（8/17）和43.8%（14/32），差异无统计学意义（*P*=0.825）。免疫单药与免疫联合化疗组的中位PFS分别为8.0个月和6.8个月，差异无统计学意义（*P*=0.502）。并对本组PD-L1高表达患者免疫治疗的预测因素进行了分析，结果显示，一线免疫治疗ORR（12/19, 63.2%）显著优于二线及以上免疫治疗（10/30, 33.3%），差异有统计学意义（*P*=0.041），二者间PFS无差异。年龄、性别、吸烟史、功能状态评分（performance status, PS）、病理类型、肿瘤大小、肿瘤淋巴结转移（tumor node metastasis, TNM）分期与ORR和PFS不相关。

**结论:**

PD-L1高表达的晚期NSCLC患者接受免疫单药和免疫联合化疗的疗效相近。PD-L1高表达患者一线免疫治疗的ORR更佳。对此类人群的最佳治疗方案有待于前瞻性临床研究进一步探索。

## Abstract (en)

**Background and objective:**

Immunotherapy represented by immune checkpoint inhibitors (ICIs) has been widely used in the treatment of lung cancer. There are controversies in clinical practice for patients with advanced non-small cell lung cancer (NSCLC) and high programmed cell death-ligand 1 (PD-L1) expression receiving ICIs monotherapy or combination chemotherapy.

**Methods:**

This study retrospectively analyzed the clinical data of 49 patients with advanced NSCLC and high PD-L1 expression. Immunohistochemistry was performed with 22C3 antibody, and the expression level of PD-L1 was evaluated according to tumor proportion score (TPS). Objective response rate (ORR) and progression free survival (PFS) were compared by groups of different clinical characteristics.

**Results:**

ORR of monotherapy and combination therapy group was 47.1% (8/17) and 43.8% (14/32), respectively, without statistical difference (*P*=0.825). The median PFS of monotherapy and combination therapy group was 8.0 months and 6.8 months, respectively, without statistical difference (*P*=0.502). Statistical analysis of predictors of immunotherapy for the patients showed first-line immunotherapy had better ORR than subsequent immunotherapy (12/19, 63.2% *vs* 10/30, 33.3%, *P*=0.041), however no difference in PFS. And there were no differences in ORR or PFS among groups of age, gender, smoking status, performance status (PS), pathological type, tumor size and tumor-node-metastasis (TNM) stage.

**Conclusion:**

The therapeutic effect is similar between ICIs monotherapy and combination chemotherapy for patients with advanced NSCLC and high PD-L1 expression. ORR of first-line immunotherapy was better in patients with advanced NSCLC and high PD-L1 expression. The optimal treatment for this population remains further prospective clinical studies.

肺癌是发病率和死亡率增长最快，对人群健康和生命威胁最大的恶性肿瘤之一。非小细胞肺癌（non-small cell lung cancer, NSCLC）约占所有肺癌的85%以上^[1]^，晚期NSCLC患者5年生存率仅为5%。以免疫检查点抑制剂（immune checkpoint inhibitors, ICIs）为代表的免疫治疗极大地改变了晚期NSCLC的治疗模式和预后。免疫治疗可将晚期NSCLC患者的5年生存率由5%提升至总体15.5%-23.2%，程序性死亡受体配体1（programmed cell death ligand 1, PD-L1）高表达，即肿瘤比例评分（tumor proportion score, TPS）≥50%的患者5年生存率可达25%-29.6%^[2]^。KEYNOTE-024^[3]^、KEYNOTE-042^[4]^、KEYNOTE-010^[5]^、IMpower110^[6]^等多项临床试验表明，对于PD-L1高表达晚期NSCLC患者，ICIs单药疗效优于化疗。同样，也有KEYNOTE-189^[7]^、KEYNOTE-407^[8]^和IMpower131^[9]^等临床试验证实，对于PD-L1高表达晚期NSCLC患者，免疫联合化疗的疗效优于化疗。因此，美国国立综合癌症网络（National Comprehensive Cancer Network, NCCN）指南（2020v6）推荐，对于PD-L1高表达（TPS≥50%）且表皮生长因子受体（epidermal growth factor receptor, *EGFR*）、间变性淋巴瘤激酶（anaplastic lymphoma kinase, *ALK*）、原癌基因1酪氨酸激酶（c-ros oncogene 1 receptor kinase, *ROS1*）、鼠类肉瘤病毒癌基因同源物B1（v-raf murine sarcoma viral oncogene homolog B1, *BRAF*）、间质-上皮细胞转化因子（mesenchymal-epithelial transition factor, *MET*）、原癌基因酪氨酸蛋白激酶受体（proto-oncogene tyrosine-protein kinase receptor, *Ret*）等驱动基因阴性及无程序性死亡受体1（programmed cell death protein 1, PD-1）/PD-L1单抗禁忌的NSCLC患者，一线首选帕博利珠单抗或铂类+培美曲塞+帕博利珠单抗（或阿特珠单抗）^[4]^，均为Ⅰ类证据。

对于PD-L1高表达的晚期NSCLC患者，临床试验证据中仅有单纯免疫治疗与化疗的比较、免疫治疗联合化疗与化疗的比较，但是，目前尚无单纯免疫治疗与免疫治疗联合化疗疗效的循证医学证据。因此，对于PD-L1高表达的晚期NSCLC患者，采用单药免疫治疗还是免疫联合化疗在临床上仍存争议。本研究通过收集我院接受ICIs治疗的PD-L1高表达晚期NSCLC患者的临床资料，比较真实世界中单药免疫治疗和免疫联合化疗的疗效。

## 1 资料与方法

### 1.1 患者筛查标准

回顾性收集2017年8月-2020年5月在北京胸科医院接受ICIs治疗的NSCLC患者的诊疗资料。纳入标准：①组织病理确诊为Ⅲb期或Ⅳ期NSCLC；②免疫组化（22C3, Dako）检测PD-L1表达≥50%；③接受ICIs治疗；④无严重的重要脏器合并症；⑤无活动性自身免疫性疾病。排除标准：①双原发肿瘤；②无可评价病灶；③无疗效评价信息。

### 1.2 PD-L1检测

组织经过10%中性福尔马林固定12 h-72 h，常规脱水透明浸蜡及石蜡包埋，每例标本连续切片3张（其中最后1张切片做HE染色确认组织形态及肿瘤细胞数量），切片厚4 μm，防脱载玻片捞片。将白片（60±2）℃烤60 min，置于二甲苯脱蜡，梯度乙醇脱水，进行抗原修复，蒸馏水漂洗后使用22C3抗体（PD-L1 IHC 22C3 pharmDx, Dako）进行免疫组织化学染色，常规脱水、透明、封片。结果判读采用TPS，定义是指部分或完整膜染色（≥1+）的肿瘤细胞占样品中存在的所有活肿瘤细胞（阴性和阳性）的百分比。两名高年资病理医师采用双盲判读，结果不一致者，重新阅片；结果相差较明显者，一起镜下阅片确定评分。

### 1.3 资料收集

从医院电子病历系统记录患者临床诊疗资料，包括人口学特征、吸烟史、功能状态评分（performance status, PS）、肿瘤病理类型、肿瘤大小、临床分期、PD-L1表达水平、免疫治疗方式以及免疫治疗线数等。

### 1.4 疗效评估及随访

患者治疗前及治疗期间（每6周）进行影像学检查（颅脑核磁、胸部计算机断层扫描（Computed tomography, CT）、腹部CT、骨扫描、浅表淋巴结超声等）。疗效评价采用实体瘤疗效评估标准1.1版（Response Evaluation Criteria in Solid Tumors, RECIST v1.1）。通过患者定期入院及电话咨询方式随访，末次随访时间为2020年10月31日。中位无进展生存时间（median progression free survival, mPFS）定义为接受免疫治疗开始到观察到疾病进展或者因任何原因死亡的时间。总生存期（overall survival, OS）定义为接受免疫治疗开始到因任何原因死亡的时间。

### 1.5 统计分析

所有统计分析均采用SPSS 24.0软件，使用χ^2^检验或*Fisher*精确检验进行率的比较；使用*Kaplan-Meier*方法进行生存分析，*Log-rank*检验进行组间比较。*P*<0.05为差异有统计学意义，*P*值均为双侧检验。

## 2 结果

### 2.1 患者特征

共有49例患者符合纳入与排除标准。表 1为49例NSCLC患者的基本特征，中位年龄64岁（35岁-80岁）。65岁以下30例（61.2%），65岁及以上19例（38.8%）；男性43例（87.8%），女性6例（12.2%），吸烟者41例（83.7%），不吸烟者8例（16.3%）；PS评分0分-1分46例（93.9%），2分3例（6.1%）；腺癌29例（59.2%），鳞癌20例（40.8%）；Ⅲb期5例（10.2%），Ⅳ期44例（89.8%）；肿瘤最大径平均4.6 cm， < 5 cm的有29例（59.2%），≥5 cm的有20例（40.8%）；免疫单药治疗17例（34.7%），免疫联合化疗32例（65.3%）；一线免疫治疗19例（38.8%），二线及以上免疫治疗30例（61.2%）。29例腺癌患者均行基因检测，7例*EGFR*突变，8例*KRAS*突变，其余14例驱动基因阴性；*EGFR*突变患者免疫治疗前均经过规范靶向治疗；*KRAS*突变患者中有4例采用一线免疫治疗，4例免疫治疗前接受化疗。

**1:** 49例NSCLC患者的临床特征 Clinicopathological features of 49 NSCLC patients

Category		*n* (%)
Age (yr)	< 65 ≥65	30 (61.2) 19 (38.8)
Gender	Male Female	43 (87.8) 6 (12.2)
Smoking status	Yes No	41 (83.7) 8 (16.3)
Performance status (ECOG)	0-1 2	46 (93.9) 3 (6.1)
Pathological type	Adenocarcinoma Squamous cell carcinoma	29 (59.2) 20 (40.8)
Tumor size (cm)	< 5 ≥5	29 (59.2) 20 (40.8
TNM stage	Ⅲb Ⅳ	5 (10.2) 44 (89.8)
Immunotherapy protocols	Monotherapy Combination therapy	17 (34.7) 32 (65.3)
Immunotherapy lines	First-line therapy Subsequent therapy	19 (38.8) 30 (61.2)
ECOG: Eastern Cooperative Oncology Group; TNM: tumor-node-metastasis; NSCLC: non-small cell lung cancer.		

### 2.2 客观缓解率（objective response rate, ORR）

全组ORR为44.9%。根据临床特征分组的ORR结果：65岁以下36.7%，65岁及以上57.9%；男性48.8%，女性16.7%；吸烟者51.2%，不吸烟者12.5%；PS 0分-1分者45.7%，PS 2分者33.3%；腺癌37.9%，鳞癌55%；肿瘤最大径 < 5 cm 37.9%，肿瘤最大径≥5 cm 55%；Ⅲb期60%，Ⅳ期43.2%；免疫单药治疗47.1%，免疫联合化疗43.8%；一线免疫治疗63.2%，二线及以上免疫治疗33.3%。χ^2^检验显示，单纯免疫治疗与免疫联合化疗的ORR差异无统计学意义（*P*=0.825）。一线免疫治疗与二线及以上免疫治疗的ORR差异有统计学意义（*P*=0.041）（表 2）。

**2:** 不同临床特征与客观缓解率的关系 Relationship between different clinical features with ORR

Category		CR+PR	SD+PD	*χ*^2^	*P*
Age (yr)	< 65	11	19	2.119	0.145
	≥65	11	8		
Gender	Male	21	22	-	0.204^*^
	Female	1	5		
Smoking status	Smoker	21	20	-	0.059^*^
	Non-smoker	1	7		
Performance status (ECOG)	0-1	21	25	-	1.000^*^
	2	1	2		
Pathological type	Adenocarcinoma	11	18	1.394	0.238
	Squamous cell carcinoma	11	9		
Tumor size (cm)	< 5	11	18	1.394	0.238
	≥5	11	9		
TNM stage	Ⅲb	3	2	-	0.646^*^
	Ⅳ	19	25		
Immunotherapy protocols	Monotherapy	8	9	0.049	0.825
	Combination therapy	14	18		
Immunotherapy lines	First-line therapy	12	7	4.182	0.041
	Subsequent therapy	10	20		
ORR: objective response rate; CR: complete response; PR: partial response; SD: stable disease; PD: progressive disease. ^*^:* Fisher* exact test.					

### 2.3 生存分析

全组49例PD-L1高表达的NSCLC患者的中位随访时间为12.5个月（1.4个月-22.5个月），35例疾病进展（71.4%），中位PFS为7个月，死亡19例（38.8%）。依据临床特征分组的中位PFS：65岁以下7.1个月，65岁及以上7个月；男性7.1个月，女性4.5个月；吸烟者7.1个月，不吸烟者4.5个月；PS 0分-1分7.6个月，PS 2分5.1个月；腺癌7.1个月，鳞癌6.8个月；肿瘤最大径 < 5 cm 7.6个月，肿瘤最大径≥5 cm 4.6个月；Ⅲb期8个月，Ⅳ期6.8个月；免疫单药治疗8个月，免疫联合化疗6.8个月；一线免疫治疗7个月，二线及以上免疫治疗7.1个月。*Log-rank*检验显示差异均无统计学意义（表 3）。免疫单药治疗组与免疫联合化疗组的生存曲线见图 1。

**3:** 不同临床特征与无进展生存期的关系 Relationship between different clinical features with PFS

Category		mPFS (mon)	95%CI	*P*
Age (yr)	< 65	7.1	4.365-9.835	0.962
	≥65	7.0	3.514-10.486	
Gender	Male	7.1	4.761-9.439	0.725
	Female	4.5	1.499-7.501	
Smoking status	Smoker	7.1	4.726-9.474	0.834
	Non-smoker	4.5	1.530-7.470	
Performance status (ECOG)	0-1	7.6	5.101-10.099	0.101
	2	5.1	0.000-12.622	
Pathological type	Adenocarcinoma	7.1	3.465-10.735	0.563
	Squamous cell carcinoma	6.8	4.875-8.725	
Tumor size (cm)	< 5	7.6	6.221-8.979	0.088
	≥5	4.6	1.486-7.714	
TNM stage	Ⅲb	8.0	0.552-15.448	0.223
	Ⅳ	6.8	4.469-9.131	
Immunotherapy protocols	Monotherapy	8.0	4.304-11.696	0.502
	Combination therapy	6.8	4.597-9.003	
Immunotherapy lines	First-line therapy	7.0	0.000-14.998	0.340
	Subsequent therapy	7.1	3.093-11.107	
mPFS: median progression free survival; CI: confidence interval.				

**1:**
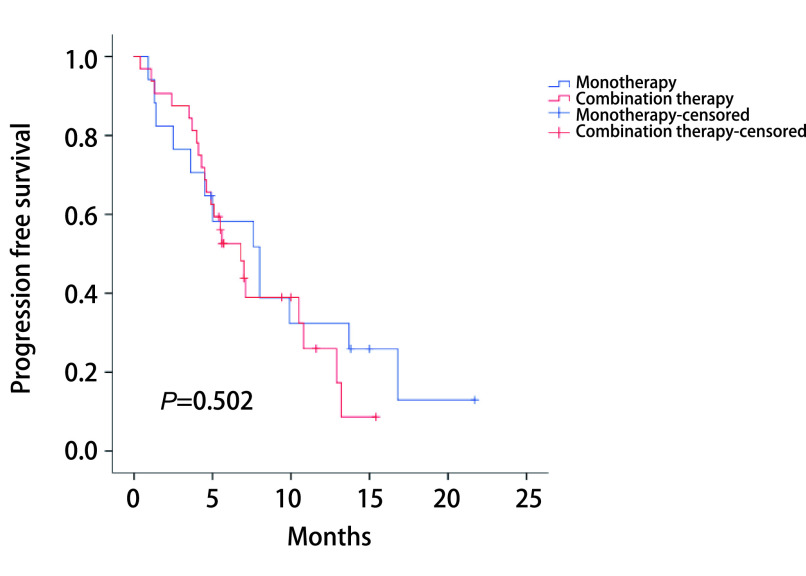
不同用药方式分组患者的无进展生存期 Progression free survival of patients with different immunotherapy protocols

## 3 讨论

我们对北京胸科医院接受免疫治疗的PD-L1高表达晚期NSCLC患者进行了免疫单药治疗和免疫联合化疗的疗效比较。结果显示，接受免疫单药治疗与免疫联合化疗患者的ORR和PFS无差异。对PD-L1高表达免疫治疗影响因素分析显示，性别、年龄、吸烟史、PS评分等因素与ORR无关，但是治疗疗效线数与ORR相关，一线免疫治疗ORR优于二线及以上（63.2% *vs* 33.3%, *P*=0.041）。对PFS进行*Kaplan-Meier*生存分析，接受免疫单药治疗与免疫联合化疗患者的PFS差异无统计学意义。

迄今为止，尚无在PD-L1高表达晚期NSCLC人群中比较单纯免疫治疗与免疫联合化疗疗效的前瞻性临床研究，仅有两项*meta*分析中包含了PD-L1高表达人群的亚组分析结果。一项*meta*分析比较了帕博利珠单抗单药治疗和帕博利珠单抗联合化疗的5项临床试验1, 289例患者数据，间接比较PD-L1≥50%亚组患者的数据发现，帕博丽珠单抗联合化疗的ORR和PFS优于帕博丽珠单抗单药（RR=1.62, 95%CI: 1.18-2.23, *P*=0.003）和PFS（HR=0.55, 95%CI: 0.32-0.97, *P*=0.037），但两组OS差异无统计学意义（HR=0.76, 95%CI: 0.51-1.14, *P*=0.184）^[10]^。另一项*meta*分析纳入了11项随机对照临床试验的6, 731例患者信息，间接比较PD-L1≥50%亚组的患者数据发现：免疫联合化疗PFS（HR=0.54, 95%CI: 0.35-0.82）优于免疫单药治疗，OS（HR=0.86, 95%CI: 0.65-1.14）差异不显著^[11]^。两项*meta*分析研究得出了类似的结论，对于PD-L1高表达的患者，免疫联合化疗可以带来PFS的获益，但OS无获益。我们的研究结果发现在PD-L1高表达人群中，单纯免疫治疗与免疫联合化疗的疗效相近，与两项*meta*分析结果有所不同。我们分析原因如下：两项*meta*分析并非直接针对PD-L1高表达人群进行免疫单药与免疫联合化疗的比较，而为间接比较；人群和治疗方式也存在较多差异。因此两项*meta*分析结果并不能起到很强的循证医学证据。受限于医保报销政策等问题，我们研究中的人群免疫治疗药物种类较多，单纯免疫治疗患者并非都是帕博丽珠单抗，此外，本组的例数偏少。

本组数据发现，一线免疫疗ORR显著优于二线免疫治疗（63.2% *vs* 33.3%, *P*=0.041），与靶向治疗不同，靶向治疗无论在一线或后线治疗中的疗效无差异。免疫治疗的机制是通过恢复T细胞的功能杀伤肿瘤，因此的免疫状态对免疫治疗疗效有明显影响。化疗通常对机体免疫功能有抑制作用。因此，提示临床上在驱动基因阴性患者中免疫治疗可优先使用。

全组患者均采用22C3抗体进行组织的PD-L1检测。PD-L1表达水平是唯一被批准用于选择免疫治疗患者的伴随诊断生物标志物。虽然存在检测方法不一致、判读标准不统一以及肿瘤异质性影响等问题，但是肿瘤组织PD-L1表达水平仍是目前可预测ICIs的疗效的较好标志物，NCCN指南推荐在晚期NSCLC患者中除了基因检测外，还需进行免疫组化法PD-L1检测^[12]^。

免疫联合化疗的初衷主要是为了解决ICIs在人群中有效率低的问题，有观点^[13]^认为，化疗药物可以看作一种“疫苗”，它杀死肿瘤细胞导致肿瘤抗原的暴露释放，同时化疗药物对免疫系统也产生整体影响，包括活化DC细胞，激活自然杀伤细胞（natural killer cell, NK）细胞，重塑肿瘤相关巨噬细胞（tumor-associated macrophages, TAMs）细胞，降低Treg细胞活性等，理论上，免疫联合化疗可以达到1+1 > 2的效果。我们研究的不足是，样本量偏小，方案不是很统一，线数不同，此外本组患者的OS数据还不成熟。但通过真实世界的数据进行分析，对临床实践具有一定的提示。

综上所述，我们通过对真实世界的数据分析，发现PD-L1高表达的晚期NSCLC患者接受免疫单药和免疫联合化疗的疗效相近，一线免疫治疗ORR显著优于二线免疫治疗。临床医生对于PD-L1高表达晚期NSCLC患者的治疗策略，应当综合考虑患者的临床特征，包括PS评分、年龄、肿瘤负荷及基因状况等因素。在肿瘤精准治疗的时代，对于PD-L1高表达、无*EGFR/ALK*等驱动基因突变的晚期NSCLC患者，有必要开展前瞻性临床试验，探索此类患者的最佳治疗模式。

## References

[1] Siegel RL, Miller KD, Jemal A (2020). Cancer statistics, 2020. CA Cancer J Clin.

[2] Garon EB, Hellmann MD, Rizvi NA (2019). Five-year overall survival for patients with advanced non small-cell lung cancer treated with pembrolizumab: Results from the phase I KEYNOTE-001 study. J Clin Oncol.

[3] Reck M, Rodriguez-Abreu D, Robinson AG (2016). Pembrolizumab versus chemotherapy for PD-L1-positive non-small-cell lung cancer. N Engl J Med.

[4] Mok TSK, Wu YL, Kudaba I (2019). Pembrolizumab versus chemotherapy for previously untreated, PD-L1-expressing, locally advanced or metastatic non-small-cell lung cancer (KEYNOTE-042): a randomised, open-label, controlled, phase 3 trial. Lancet.

[5] Herbst RS, Garon EB, Kim DW (2020). Long-term outcomes and retreatment among patients with previously treated, programmed death-ligand 1 positive, advanced nonsmall-cell lung cancer in the KEYNOTE-010 study. J Clin Oncol.

[6] Spigel D, de Marinis F, Giaccone G (2019). IMpower110: Interim overall survival (OS) analysis of a phase Ⅲ study of atezolizumab (atezo) *vs* platinum-based chemotherapy (chemo) as first-line (1L) treatment (tx) in PD-L1-selected NSCLC. Ann Oncol.

[7] Gadgeel S, Rodriguez-Abreu D, Speranza G (2020). Updated analysis from KEYNOTE-189: Pembrolizumab or placebo plus pemetrexed and platinum for previously untreated metastatic nonsquamous non-small-cell lung cancer. J Clin Oncol.

[8] Paz-Ares L, Vicente D, Tafreshi A (2020). A randomized, placebo-controlled trial of pembrolizumab plus chemotherapy in patients with metastatic squamous NSCLC: Protocol-specified final analysis of KEYNOTE-407. J Thorac Oncol.

[9] Jotte R, Cappuzzo F, Vynnychenko I (2020). Atezolizumab in combination with Carboplatin and Nab-Paclitaxel in advanced squamous NSCLC (IMpower131): Results from a randomized phase Ⅲ trial. J Thorac Oncol.

[10] Zhou Y, Lin Z, Zhang X (2019). First-line treatment for patients with advanced non-small cell lung carcinoma and high PD-L1 expression: pembrolizumab or pembrolizumab plus chemotherapy. J Immunother Cancer.

[11] Liang H, Liu Z, Cai X (2019). PD-(L)1 inhibitors *vs.* chemotherapy *vs.* their combination in front-line treatment for NSCLC: An indirect comparison. Int J Cancer.

[12] Gadgeel SM, Stevenson J, Langer CJ (2016). Pembrolizumab (pembro) plus chemotherapy as front-line therapy for advanced NSCLC: KEYNOTE-021 cohorts A-C. J Clin Oncol.

[13] Tan D (2019). ES01.03 Chemotherapy directly targets the immune system to improve efficacy. J Thorac Oncol.

